# Subcellular compartmentalization of PKM2 identifies anti-PKM2 therapy response *in vitro* and *in vivo* mouse model of human non-small-cell lung cancer

**DOI:** 10.1371/journal.pone.0217131

**Published:** 2019-05-23

**Authors:** Akiko Suzuki, Sachin Puri, Pamela Leland, Ankit Puri, Tarsem Moudgil, Bernard A. Fox, Raj K. Puri, Bharat H. Joshi

**Affiliations:** 1 Center for Biologics Evaluation & Research, Food Drug Administration, Bethesda, Maryland, United States of America; 2 Molecular & Tumor Immunology, Robert W. Franz Cancer Research Center, Earle A. Chiles Research Institute, Providence Cancer Center, Portland, Oregon, United States of America; 3 Department of Molecular Microbiology and Immunology, OHSU, Portland, Oregon, United States of America; University of South Alabama Mitchell Cancer Institute, UNITED STATES

## Abstract

Pyruvate kinase M2 (PKM2) is an alternatively spliced variant, which mediates the conversion of glucose to lactate in cancer cells under normoxic conditions, known as the Warburg effect. Previously, we demonstrated that *PKM2* is one of 97 genes that are overexpressed in non-small-cell lung cancer (NSCLC) cell lines. Herein, we demonstrate a novel role of subcellular PKM2 expression as a biomarker of therapeutic response after targeting this gene by shRNA or small molecule inhibitor (SMI) of PKM2 enzyme activity *in vitro* and *in vivo*. We examined two established lung cancer cell lines, nine patients derived NSCLC and three normal lung fibroblast cell lines for PKM2 mRNA, protein and enzyme activity by RT-qPCR, immunocytochemistry (ICC), and Western blot analysis. All eleven NSCLC cell lines showed upregulated PKM2 enzymatic activity and protein expression mainly in their cytoplasm. Targeting PKM2 by *shRNA* or SMI, NSCLC cells showed significantly reduced mRNA, enzyme activity, cell viability, and colony formation, which also downregulated cytosolic PKM2 and upregulated nuclear enzyme activities. Normal lung fibroblast cell lines did not express PKM2, which served as negative controls. PKM2 targeting by SMI slowed tumor growth while gene-silencing significantly reduced growth of human NSCLC xenografts. Tumor sections from responding mice showed >70% reduction in cytoplasmic PKM2 with low or undetectable nuclear staining by immunohistochemistry (IHC). In sharp contrast, non-responding tumors showed a >38% increase in PKM2 nuclear staining with low or undetectable cytoplasmic staining. In conclusion, these results confirmed PKM2 as a target for cancer therapy and an unique function of subcellular PKM2, which may characterize therapeutic response to anti-PKM2 therapy in NSCLC.

## Introduction

Lung cancer is the most common cause of cancer related mortality worldwide, accounting for approximately 1 in 4 cancer deaths [1, 2]. About 85–90% of lung cancers are non-small-cell-lung cancer [3, 4]. For early stage Non-Small-Cell Lung Cancer (NSCLC), surgery is usually the treatment of choice and chemotherapy (sometimes in combination with radiation therapy) may be given as well. Patients with advanced-stage NSCLC are usually treated with chemotherapy, targeted drugs (or a combination of the two), or immunotherapy. Considering the low 5-year survival rate (21%) with currently available therapies, there is a need for improved treatment options [4]. Compared to normal cells, cancer cells display a radical shift in metabolism becoming highly dependent on glucose, which is metabolized through an increased rate of aerobic glycolysis, a metabolic state termed the Warburg effect, which is considered a hallmark of cancer metabolism [5, 6]. Previously, we have demonstrated that human NSCLC cell lines overexpress 97 genes by DNA microarray [7–9]. Among these, pyruvate kinase M2 (PKM2) is highly overexpressed in NSCLC cell lines examined compared to normal lung tissues. PKM2 is an allosteric isoform of pyruvate kinase, which catalyzes the final step in glycolysis and converts phosphoenol-pyruvate (PEP) to pyruvate [10]. PKM2 is shown to divert glycolytic flux into the pentose phosphate pathway associated with attenuated pyruvate kinase activity, thereby meeting the biosynthetic demands for rapid proliferation [10]. Of four isoforms of pyruvate kinase L, R, M1 and M2, proliferating embryonic and tumor cells predominantly express PKM2. In cancer cells, PKM2 can migrate to the nucleus and function as a transcriptional co-factor in response to many extracellular signals such as Epidermal growth factor (EGF) and hypoxia, which activate CYCLIN D1, C-MYC or Hypoxia inducible factor-alpha (HIF-α) [11, 12]. PKM2 is shown to mediate epithelial to mesenchymal transition (EMT), which stimulates PKM2 to migrate to nucleus in cancer cells and acts as a transcription cofactor that in turn inhibits E-cadherin [13]. It is also shown that cytosolic PKM2 is associated with Epidermal growth factor receptor (EGFR) expression and prolongs the protein half-life of EGFR in cancer cells by stabilizing EGFR-Heat shock protein 90 (HSP90) protein complex [14]. PKM2 is reported to act as a protein kinase and exist as a dimer localized in the nucleus promoting cell proliferation, while its tetramer form is an active pyruvate kinase localized in the cytosol [15]. It is also reported that nuclear translocation of PKM2 supports cancer cell survival, which binds to Oct4 promoting expression of cancer stemness-related genes [16].

Targeting colorectal cancer cell lines with gefitinib (a tyrosine kinase inhibitor) or oxaliplatin revealed that nuclear PKM2 contributed to therapeutic resistance *in vitro* and *in vivo* models of colorectal cancer [17, 18]. Further, it is reported that selective inhibition of PKM2 isoform with small molecules and RNAi reduced cell proliferation in human lung cancer cells *in vitro* [19]. As many oncogenes can influence glucose metabolism in cancer cells, inhibition of the PKM2 isoform may have a variable degree of therapeutic applicability for *in vitro* and *in vivo* models of human cancers. Silencing *PKM2* specific RNA decreased cell viability and increased apoptosis in several cancer cell lines *in vitro* and regressed tumor volumes by delivering them in *in vivo* mouse models of hepatocellular and ovarian cancer [20]. Additionally, PKM2 also acts as a protein kinase in the nucleus upon EGFR activation, hypoxia or glucose depletion, where it can enhance the proliferation and survival of certain types of cancer cells in response to mitogen activation or hypoxic stress [19, 21–23]. Previous studies have shown that PKM2 can act as one of the downstream transcriptional coactivators of EGF/EGFR signal transduction, which promotes disease progression in glioma cells and it might be upregulated through the EGFR-dependent NF-kB activation [23, 24]. As PKM2 inhibits P53-dependent transactivation of the P21 gene by preventing P53 binding to the P21 promoter, it may lead to increased G1 phase activity, thus providing a growth advantage for tumor cells in the presence of a DNA damage stimulus [25]. PKM2 isoform is expressed universally in many types of rapidly growing cells such as embryonic cells and cancer cells, which supports cancer growth by regulating a group of genes involved in cell proliferation, migration and apoptosis [26].

Because patient-derived and established NSCLC cell lines showed overexpression of PKM2 and PKM2 enzyme activities, we hypothesized that inhibiting enzyme activity by small molecule inhibitor (SMI) or gene silencing by shRNA (*shRNA-PKM2)* might influence NSCLC growth kinetics. Here, we tested whether SMI and *shRNA-PKM2* influence NSCLC growth kinetics *in vitro* and *in vivo*. Our results demonstrate that PKM2 is primarily expressed in the cytosol of NSCLC cells and xenograft tumor cells. Both approaches (SMI and *shRNA-PKM2*) not only inhibited the PKM2 enzyme activity and cell proliferation in NSCL cells, but also decreased PKM2 mRNA expression and ultimately led to cell killing by apoptosis. Additionally, our results showed a unique subcellular compartmentalization of PKM2 in NSCLC cells and xenografts upon treating them with anti-PKM2 therapies *in vitro* and *in vivo* indicating that PKM2 expression may serve as a biomarker of therapeutic response in NSCLC.

## Materials and methods

### Cell lines and treatment

We studied nine patient-derived NSCLC cell lines, which were previously established at the Earle A. Chiles Research Institute, Portland, OR. The two cell lines established at the NCI (H1299 and H358) and 3 normal lung fibroblast cell lines (WI-38, HEL-299 and IMR-90) were purchased from ATCC and were maintained in the ATCC recommended complete medium. These cells lines were purchased from ATCC within first two years of undertaking experiments and if not their identity was confirmed by short-tandem repeats (STR) profiling. The nine cell lines from the Earle A. Chiles Research Institute were cultured in complete medium with 10% FBS. A small molecule inhibitor (SMI) of the PKM2 enzyme inhibitor (SMI-Compound 2825–0090, N-(3-carboxy-4-hydroxy) phenyl 1–2,5,-dimethylpyrole) was purchased from ChemDiv (San Diego, CA) and dissolved in PBS for treating the NSCLC and normal lung cell lines. For PKM2 targeting in NSCLC cell lines, *shRNA* (AGAGCCTACCTGTATGTCAAT) and control plasmids (AGAGCCTACCTGTATGTCAAT) were purchased from Qiagen (Qiagen, Germantown, MD) and transfected by using Attractene transfection reagent (Qiagen) as suggested by the manufacturer.

### RT-qPCR

Total RNA from 3 normal lung and 11 NSCLC cell lines was extracted after incubating them with different concentrations of PKM2 inhibitor (1 to 80 μM) for 48 hours at 37°C in a CO_2_ incubator using RNA-easy mini prep kit (Qiagen) following the manufacturer’s instructions and amplified using forward; CTATCCTCTGGAGGCTGTGC and backward; CCAGACTTGGTGAGGACGAT primers for human PKM2. β-actin gene specific primers (forward: TGAGAGGGAAATCGTGCGTG and backword TGC TTG CTG ATC CAT ACC ATC TGC) were used to amplify β-actin as a house keeping gene for quantitative expression of PKM2 mRNA [27, 28]. Briefly, 100ng of total RNA was reverse transcribed by using one step RT-qPCR kit using CFX 96 Real time detection system (Bio-Rad, Hercules, CA) using gene specific primers (Bio-Rad, Hercules, CA) as recommended by the manufacturer. Each sample was run in triplicate and the data were expressed as % ratio of relative fluorescence units of PKM2and β -actin.

### Pyruvate kinase activity

Pyruvate kinase activity in control, SMI-treated or shRNA-transfected NSCLC cells was measured by a continuous assay coupled to lactate dehydrogenase (LDH). To assess activity in cell lysates, cells were treated with SMI for the indicated time and lysed in NP40 containing 25 μM Tris/Cl pH 7.4 lysis buffer immediately before measuring pyruvate kinase activity as described previously [10, 29]. Briefly, the change in absorbance at 340 nm, caused by oxidation of NADH, was measured using Ultrospec 2000 spectrophotometer (Pharmacia/LKB,). The assay was performed in triplicate from each sample lysate and values were expressed as % normal lung cell line lysates.

### Cell viability assay

Cell viability of NSCLC cell lines was measured by trypan blue exclusion technique. The number of viable cells was manually counted using a hemocytometer and expressed as % of untreated cells [30, 31].

### Clonogenic assay

Colony formation of NSCLC cell lines in SMI-treated or *shRNA* targeted cultures was measured *in vitro* as described previously [32]. The number of colonies formed were counted and expressed as number of colonies in untreated, SMI treated or *shRNA-PKM2* transfected cell cultures.

### Assessment of apoptosis

Patient-derived and established NSCLC cell lines were seeded in 4 well glass chambered slides and incubated with 0–80 μM of SMI for 5 days. After a brief wash with 1x PBS, the TUNEL positive apoptotic cells were counted as per manufacturer’s instructions (Promega Corporation, Madison, WI). The assay was performed in triplicate and data were shown as mean ± SD. Intracellular caspase3/7 activity was measured from control (untreated), sh-negative control and *shRNA-PKM2* transfected NSCLC cells using earlytox Caspase-3/7 R100 assay kit following manufacturer’s recommendations (Molecular Device, Sunnyvale, CA). The endpoint fluorescence was measured on a SpectraMax M5 plate reader and each value is expressed as arbitrary fluorescence units.

### Immunocytochemical and immunohistochemical analysis (ICC and IHC)

PKM2 expression in NSCLC cells and xenografts sections was analyzed by ICC and IHC techniques as described previously [28, 33]. For PKM2 expression in NSCLC xenografts, the cryo-sections were immunostained with rabbit monoclonal anti-PKM2 antibody (clone EPR10138, Abcam, Cambridge, MA) and DAPI also as described above. The degrees of cytoplasmic and nuclear PKM2 fluorescence in red and nuclear staining with DAPI in blue fluorescence in sections were evaluated for subcellular compartmentalization of PKM2 expression by using Nikon-S- Element software (Nikon, Melville, NY). The extent of immunostaining was evaluated as ≤1+, negative; 2+, positive; 3+, strongly positive; 4+, more strongly positive. Three independent investigators (BJ, AS, SP) scored IHC images in a blinded fashion for subcellular localization of PKM2 levels.

### Subcellular fractionation

Twenty million NSCLC or normal lung cells were washed with 1x PBS before processing for subcellular fractionation. Nuclear and cytoplasmic fractions from NSCLC cells and xenografts were prepared using NE-PER nuclear and cytoplasmic extraction reagents (Pierce Biotechnologies, Rockford, IL, now Thermofisher Scientific, Waltham, MA). All cytoplasmic and nuclear fractions were stored at -80°C until analyzed for PKM2 or Caspase activity or Western blot analysis.

### Western blot analysis

500,000 NSCLC cells were seeded in a 10-cm tissue culture plate and treated with different concentrations of SMI when the cell confluency was between 50–60%. After 48 hours, total cell lysate, subcellular fractions (cytosol/nuclear fractions) were prepared using NE-PER reagents as described above. The supernatant from total cell lysate, nuclear and cytosol fractions were quantitated by Bio-Rad protein assay (Bio-Rad, Hercules, California). Twenty micrograms of protein equivalent lysates were boiled in 4 x SDS sample loading buffer and run on mini-Protean TGX AnyKd gel (Bio-Rad). The proteins were transferred to nitrocellulose and Western blots were performed using rabbit monoclonal anti-PKM2 antibody (AbCam, Cambridge, MA) and incubated with IR Dye 800CW near-infrared fluorescent goat anti-rabbit IgG secondary antibody (LI-COR, Lincoln, NE). The membrane was washed three times and scanned using Odyssey CLx system (LI-COR, Lincoln, NE).

### *In vivo* PKM2 targeting study

Female nude nu/nu mice aged between 6 and 7 weeks were housed in a temperature-controlled, pathogen-free animal facility with 12-hour light and dark cycles. All animal studies were conducted under approved protocol #1992–42 by the CBER Institutional Animal Care and Use Committee (ACUC) in accordance with the principles and procedures outlined in the NIH Guideline for the Care and Use of Laboratory Animals. All animal experiments were performed under ABSL-2 conditions in a facility accredited by the Association for Assessment and Accreditation of Laboratory Animal Care (AAALAC). Animals were monitored daily with increased monitoring at a minimum of twice daily during experiments. The analgesics were not used to avoid their possible interference in PKM2 signaling and translocation to tumor cell organelles and in tumor microenvironment. Euthanasia before tissue harvest was by ketamine/xylazine overdose while euthanasia due to body weight loss or termination of study was by carbon dioxide inhalation in a chamber where the carbon dioxide was from a cylinder source delivered by Euthanex equipment (Palmer, PA).

Five million H358 cells were injected subcutaneously into the right flank of each mouse. When tumors reached 5 mm in diameter, H358-tumor-bearing mice were injected with different doses of PKM2 inhibitor (0, 50, or 500 μg/kg/day) once a day on alternate days for 12 days (total 6 injections) by i.t. injection. Tumor size was measured weekly and overall sacrifice time (OST) of the animals was calculated based on the sacrifice of mice when tumors reached 2 cm in diameter, according to NIH animal guidelines. Mice were weighed at day 10 and day 25 after PKM2 inhibitor treatment. Experiments were repeated twice.

Similarly, 5x10^6^ *shRNA-PKM2 gene* silenced or negative control plasmid transfected H358 cells were implanted in six-week-old female nu/nu mice, weighing 17-20g for developing xenografts as described above. Before implanting the cells, they were either PKM2 gene silenced, mock-transfected or negative control plasmid transfected and selected by using G148 antibiotic. The transfected NSCLC cells were tested for PKM2 RNA and protein by RT-qPCR and IFA assays. For these experimental differences of transfection and selection involved in before implanting the cells in mice, the control group of mice was different compared to SMI treated mice, which were labeled as non-silenced mice in which negative control sh-RNA plasmid was used. The therapeutic response and OST were evaluated between these two groups. The xenografts in mice, which responded to anti-PKM2 therapy (either SMI or *shRNA-PKM2)* were referred to regressing tumors and those not responding were termed as non-regressing tumors.

### Statistical analysis

Statistical analysis was performed using Student’s t test for comparison between two groups or ANOVA among more than two groups of mice using Graph Pad Prism program (GraphPad Software, La Jolla, CA). Survival curves were generated by the Kaplan-Meier method and compared by using the log-rank test. The level of statistical significance was set at a P value of ≤0.05.

## Results

### PKM2 is overexpressed in NSCLC cell lines

**Fig 1:**
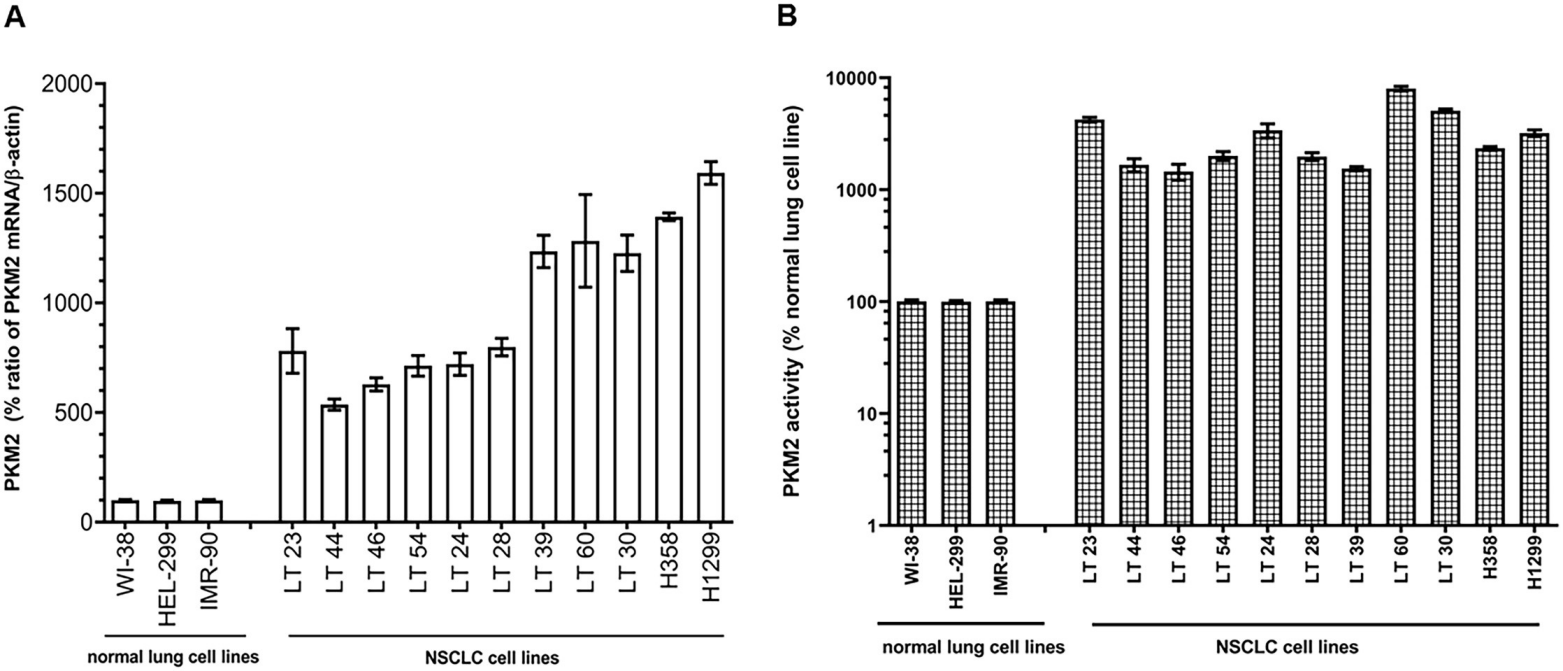
PKM2 expression in normal lung and NSCLC cell lines. (A) mRNA levels and (B) PKM2 enzyme activity were measured in 3 normal lung and 11 NSCLC cell lines. Each value is expressed as mean % of normal lung cell lines.

We observed that all patient-derived and established NSCLC cell lines significantly overexpress PKM2 mRNA ranging from 5.3 to 16.5 times greater than normal lung cell lines (P≤0.0001, Fig 1A). Further, PKM2 enzyme activity was markedly higher in all 11 NSCLC cell lysates compared to three normal lung cell lines (P≤0.0001, Fig 1B), corroborating RT-qPCR results of mRNA overexpression. We confirmed these data by analyzing all NSCLC cell lines for PKM2 protein expression by ICC using rabbit monoclonal antibody. ICC results for all these cell lines showed ≥2+ immunostaining intensity in all 11 NSCLC cell lines with 55–86% positively stained cells. In contrast, the extent of immunostaining in three normal lung cell lines was ≤ 1+ and 8–12% cells were positive for immunostaining (S1 Table and S1 Fig).

### PKM2 supports tumor growth of NSCLC cell lines

**Fig 2:**
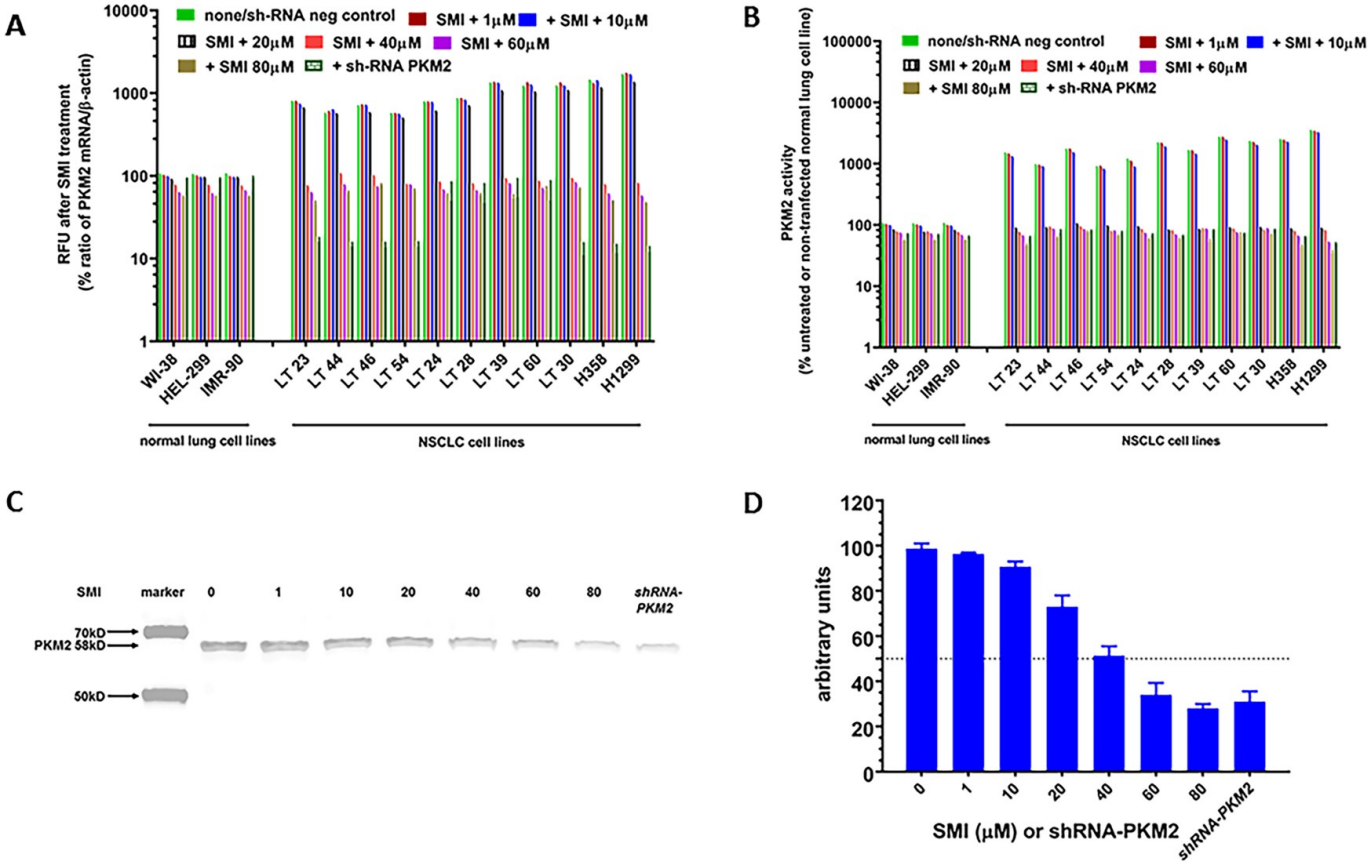
Effect of SMI on mRNA expression, enzyme activity and protein expression by Western blot analysis. Three normal lung and 11 NSCLC cell lines were treated with different concentrations of PKM2 inhibitor (10 to 80 μM). (A) Total RNA from the cells was extracted and real-time PCR was performed. Relative expression of mRNA for PKM2 and β-actin is shown as mean % of untreated normal lung cell line (Relative Fluorescence Units-RFU). (B) PKM2 enzyme activity was measured. Each value is expressed as mean % of untreated normal lung cell lines. (C) Expression of PKM2 in one representative NSCLC cell line treated with different concentration of SMI and *shRNA-PKM2* was determined by Western blot analysis. (D) Semi-quantitation of immunoblot was performed using image studio software. Dotted line indicated 50% arbitrary units of band intensity.

Based on RT-qPCR, PKM2 enzyme activities and ICC results, we hypothesized that PKM2 may have a critical role in NSCLC cell growth. As shown in Fig 2A, all NSCLC cell lines demonstrated a significant and concentration-dependent decrease in PKM2 mRNA expression after treating either with SMI for 48 hours (P = 0.0023) or PKM2 gene silencing (P = 0.001) compared to untreated (none) or negative control shRNA transfected cells. But normal lung fibroblast cell lines did not show such dramatic response to SMI treatment (Fig 2A). Further, SMI treatment and *shRNA-PKM2* resulted in a significant decrease in PKM2 enzyme activity (P<0.005 and P<0.00012) and a marginal decrease in normal lung cell lines (Fig 2B) compared to corresponding untreated control cell lines. As shown in Fig 2C, we confirmed RT-qPCR and PKM2 enzyme data by performing Western blot analysis from total cell lysates of untreated (labeled as none), treated with different concentrations of SMI and *shRNA-PKM2* cell lysates. We observed that no treatment (none) and *shRNA* negative control lysates showed similar values, and therefore pooled together in Fig 2A, 2B and 2C. Interestingly, PKM2 protein levels by Western blot corroborated the RT-qPCR and PKM2 enzyme activity (Fig 2D), which demonstrated a concentration dependent decrease in PKM2 protein levels, although none of these two approaches completely blocked PKM2 expression even at higher than 65 μM concentrations of SMI (data not shown).

**Fig 3:**
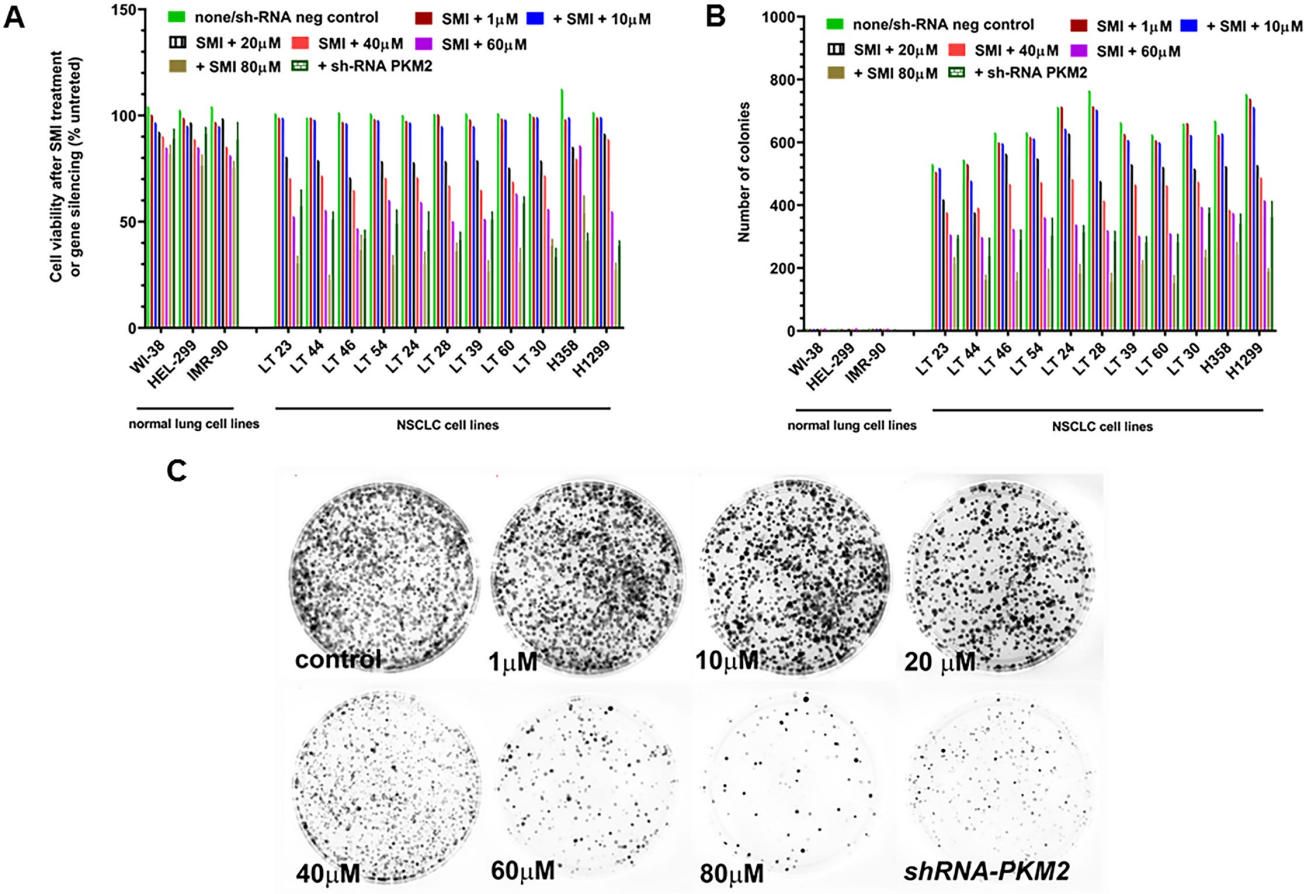
Effect of SMI on cell viability and clonogenicity of NSCLC cell lines. NSCLC cell lines were treated with different concentrations of SMI (10 to 80 μM). (A) number of viable cells were expressed as % untreated cells counted by trypan blue exclusion technique in SMI treated and *shRNA-PKM2* silenced 3 normal lung and 11 NSCLC cell lines. (B) For clonogenic assay, cells were incubated for 8 days to form colonies. Number of colonies was counted. (C) Number of colonies formed by NSCLC cells treated with different concentrations of SMI and shRNA PKM2 were stained with 0.25% crystal violet in 25% ethanol, washed with water and air dried.

Further, targeting PKM2 with SMI resulted in a concentration-dependent decrease in NSCLC cell viability where the IC_50_ of SMI (concentration of SMI causing 50% inhibition of NSCLC cell line growth) ranged between 30–65 μM (Fig 3A). At 20 μM SMI concentration, ~80% cells were viable. Normal fibroblast lung cell lines did not demonstrate sensitivity to SMI, which corroborated RT-qPCR and ICC data showing low expression of PKM2 (S2 Table). These observations were independently confirmed by testing the effect of SMI/ *shRNA-PKM2* on colony formation of NSCLC *in vitro* (Fig 3B and 3C). The number of colonies from NSCLC cells treated with SMI decreased significantly (P<0.001). Under similar SMI treatment conditions, we observed approximately 50% decrease in number of colonies at ≤65 μM SMI, which corroborated its effects on cell viability in a concentration-dependent manner in SMI treated NSCLC cultures.

To examine whether PKM2 is critical for cell viability of NSCLC cells, we analyzed cell viability in *shRNA-PKM2* transfected cell lines by trypan blue dye exclusion technique. *sh-RNA-PKM2* also caused a marked reduction in cell viability and colony formation (P<0.001; Fig 3A–3C). Indirect immunofluorescence analyses of SMI treated or *shRNA-PKM2* NSCLC cell lines revealed a significant decline in the extent of immuno-staining and the percentage of PKM2 positive cells compared to corresponding controls (S2 Table, P<0.0001).

### Targeting of PKM2 by SMI or *shRNA-PKM2* modulates subcellular PKM2 activity *in vitro*

**Fig 4:**
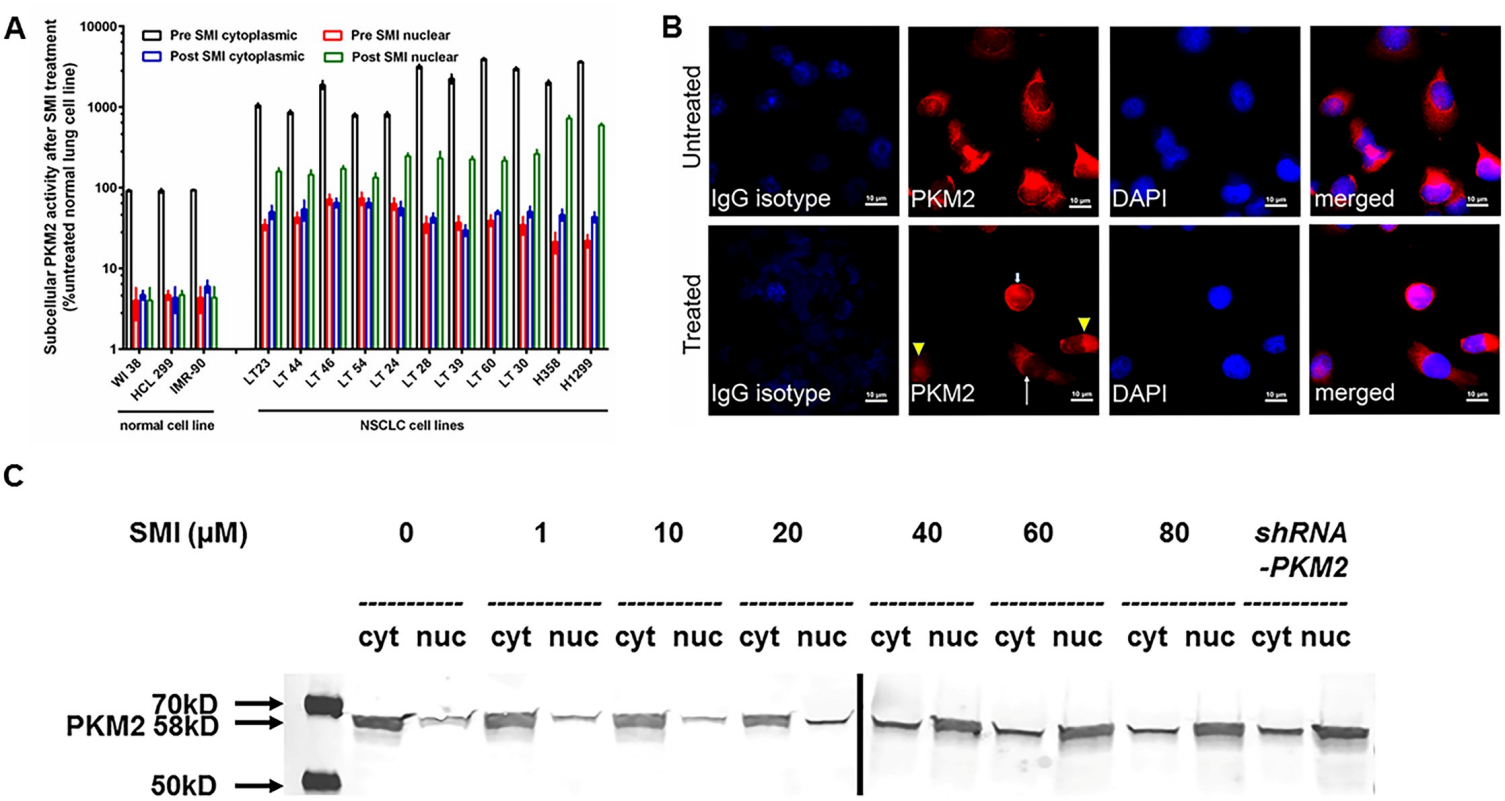
Effect of PKM2 inhibitor on subcellular localization of PKM2 enzymatic activities and protein levels. (A) Three normal lung and 11 NSCLC cell lines were incubated with 65 μM of PKM2 inhibitor for 48 hours. Cytoplasmic and nuclear fractions were separated for PKM2 enzyme activity determination. Each value is expressed as mean % cytoplasmic activity in untreated normal lung cell lines. (B) 25,000 H358 cells were plated in a multiwell-glass chamber and incubated for 48 hours with or without 65 μM of PKM2 inhibitor. Then, IFA was performed. PKM2 was stained with Alexa 594 (red). Untreated cells stained with anti-PKM2 antibody showed spindle-shaped morphology with red cytoplasm and hollow nucleus while treated cells showed various patterns of red signals; low signal in both cytoplasm and nucleus (arrow head), small cells with intense signal in both cytoplasm and nucleus (small arrow), and spindle-shaped cells with red cytoplasm and hollow nucleus (big arrow). (C) Western blot analysis for PKM2 was performed from cytoplasmic and nuclear fractions of a NSCLC cell line treated with 10–80 μM of SMI or *shRNA-PKM2*.

As PKM2 is important for NSCLC cell growth *in vitro*, we further evaluated the functional importance of subcellular PKM2 expression in terms of its subcellular localization and activity in SMI treated or *shRNA-PKM2* gene silenced NSCLC cell lines. We observed that the cytoplasmic PKM2 enzyme activity was significantly higher than nuclear enzyme activities (P<0.001) in untreated NSCLC cell cultures. In contrast, treatment of NSCLC lines with SMI for 48 hours or *sh-RNA-PKM2* resulted in a decrease in cytoplasmic enzyme activity with a concomitant rise in nuclear PKM2 enzyme activity (Fig 4A). Further, we observed that untreated cells stained with rabbit monoclonal anti-PKM2 antibody showed spindle-shaped morphology with red cytoplasm and hollow nucleus, while treated cells showed various patterns of red signals; low signal in both cytoplasm and nucleus, small and round cells with intense signal in both cytoplasm and nucleus, and spindle-shaped cells with red cytoplasm and hollow nucleus (Fig 4B). As shown in (Fig 4A), treatment of NSCLC cell line with IC_50_ concentration of SMI for 48 hours resulted in a decrease in cytoplasmic enzyme activity. By IFA analysis, we observed that for untreated NSCLC cells, anti-PKM2 antibody immunostaining identified ≥2+ staining of cytoplasmic PKM2 and ≤ 1+ staining of nuclei (Fig 4B). However, SMI treatment showed a significant decrease in immunostaining for PKM2 in the cytoplasm (≤ 1+) with a concomitant increase of immunostaining in nuclei (≥2+) of established NSCLC cell lines (Fig 4B and S3 Table). Consistent with these observations, NSCLC cell lines silenced by the *shRNA-PKM2* revealed a similar decrease in cytoplasmic PKM2 and increased expression in nuclei of NSCLC cells (S3 Table). Next, we performed Western blot analysis for PKM2 protein levels in cytoplasmic and nuclear fractions of SMI treated or *shRNA-PKM2* NSCLC cell lines. Fig 4C demonstrates a 58kDa PKM2 protein band in the immunoblot in which the cytoplasmic PKM2 protein was decreasing in a concentration dependent manner upon treatment with SMI or *shRNA-PKM2* NSCLC cell lines. In contrast, the nuclear PKM2 protein was increasing in NSCLC cell lines treated with SMI or gene silencing.

### Targeting of PKM2 by SMI or *shRNA-PKM2* induced apoptosis

**Fig 5:**
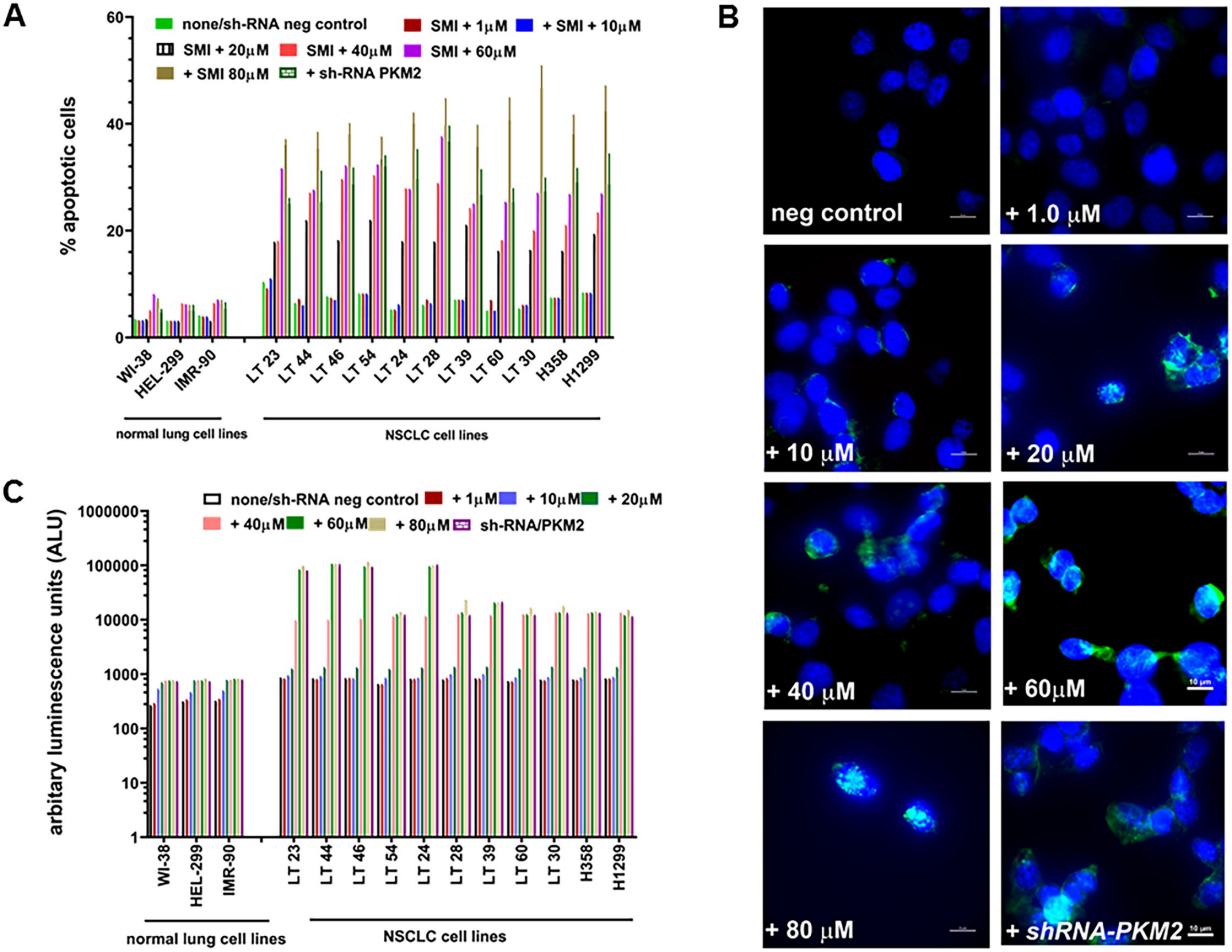
SMI and PKM2 silencing induced apoptosis in NSCLC cell lines. (A) Number of apoptotic nuclei by TUNEL assay were counted in 3 normal lung and 11 NSCLC cell lines after treating with 10–80μM of SMI or shRNA. Data are presented as mean ± SD from three independent experiments as described in Materials and Methods. (B) 1000 x magnification image of a NSCLC cell line to show apoptotic nuclei after treating with 10–80μM of SMI in TUNEL assay (C) SMI treatment induces Caspase 3/7 activity in normal lung and NSCLC cell lines in a concentration dependent manner. Data are presented as mean ± SD from three independent experiments as described in Materials and Methods.

To determine whether the effects of SMI or gene silencing on cell viability of NSCLC cell lines are dependent on induction of apoptosis, we determined if the loss of viability is mediated by Caspase 3/7 and induction of apoptosis in PKM2 depleted NSCLC and normal lung cell lines by Tunel assay. We observed a concentration-dependent increase in apoptotic cells in SMI-treated NSCLC cultures detected by the Tunel assay (Fig 5A). *shRNA-PKM2* NSCLC cell lines also showed a sharp increase in apoptotic cells compared to their negative control counterpart cultures. As shown in Fig 5B, TUNEL assay microscopic images of SMI treated or *shRNA-PKM2* NSCLC cultures demonstrated increased number of apoptotic nuclei. We also observed a significant increase in Caspase 3/7 activities (P = 0.0023) in SMI treated or *shRNA-PKM2* NSCLC cells compared to respective controls. In contrast, three normal lung fibroblast cell lines failed to show such increase in Caspase 3/7 activities (Fig 5C).

### Targeting of PKM2 by SMI or *shRNA-PKM2* inhibited tumor growth of NSCLC xenografts *in vivo*

**Fig 6:**
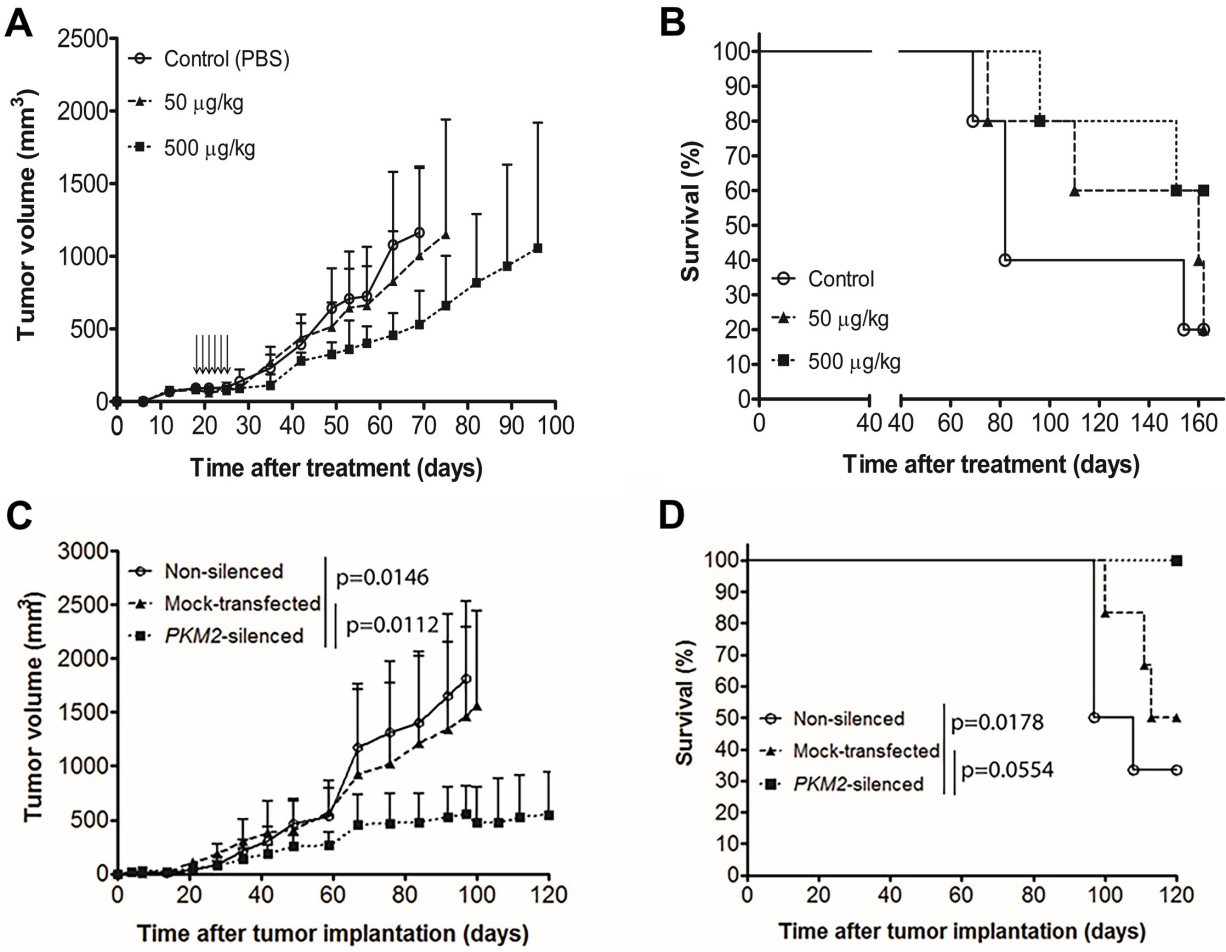
Antitumor effect of PKM2 inhibitor and *shRNA-PKM2* in NSCLC s.c. tumor models. (A) H358 tumors implanted mice were intratumorally treated with PKM2 inhibitor when tumors reached 5 mm in diameter. Tumor volume of each mouse was measured. Bars represent S.D. Each arrow indicated SMI injection to mice. On day 51, there was no significant difference between the groups. (B) Kaplan-Meier survival curves of H358 s.c. tumor-bearing mice after treatment were plotted. Overall sacrifice time (OST) was calculated based on the sacrifice of mice when tumors reached >2cm. Median survival: Control 64 days, 50μg/kg 142 days, and 500 μg/kg undefined. (C) H358 cells (non-silenced, mock-transfected, and *PKM2*-silenced) were implanted subcutaneously in nude mice and tumor volume was measured. Bars represent S.D. On day 100, there was significant difference between the groups; parental vs *PKM2*-silenced and mock-transfected vs *PKM2*-silenced (p = 0.0146 and 0.0112, respectively). (D) Kaplan-Meier survival curves of H358 s.c. tumor-bearing mice were plotted. Median survival: Non-silenced 102.5 days, mock-transfected 113.5 days, *PKM2*-silenced undefined.

To investigate whether PKM2 inhibition by SMI or *shRNA-PKM2* mediated anti-tumor effects *in vivo*, we first established xenografts using a slow growing H358 tumor cell line in athymic nude mice by implanting 5 x 10^6^ cells. When tumors reached to 62.5mm^3^, we treated mice with 50 or 500 μg/kg doses of SMI intratumorally every alternate day for a total of 6 injections. The control mice received PBS. We observed that the lowest dose SMI treatment did not inhibit tumor growth (Fig 6A). However, the higher dose of SMI inhibited tumor growth. Interestingly, the treatment of tumor bearing mice by 50 and 500 μg/kg SMI prolonged the survival by a median of >80 days compared to control mice, however, no significant difference was shown by log-rank test (Fig 6B). In contrast, *shRNA-PKM2* xenografts showed a significant reduction in tumor size over a follow up of 100 days compared to non-silenced and mock-transfected xenografts (Fig 6C, P = 0.0146 and 0.0112, respectively). A significant increase in OST in mice with PKM2 silenced xenografts was observed compared to non-silenced group (Fig 6D, P = 0.0178). However, OST between PKM2 silenced and mock-transfected control xenografts was not significant (P = 0.0554). We also studied the effects of SMI and *shRNA-PKM2* on another fast-growing xenografts tumor model implanted with H1299 NSCLC cells and observed similar results. In brief, SMI showed modest effects on tumor growth, however, *shRNA-PKM2* xenografted animals showed a significant reduction in tumor size (data not shown).

### Toxicity of PKM2 inhibitor in murine models of NSCLC

We carefully monitored the general condition and body weight of mice to evaluate the general toxicity of SMI and *shRNA-PKM2* during the course of the experiment. We observed that there was no significant difference in general appearance and body weight among four groups of animals in both tumor models (data not shown).

### Targeting of PKM2 modulates subcellular compartmentalization and identifies therapeutic response

To determine whether PKM2 inhibition modulates subcellular compartmentalization, we evaluated cytoplasmic and nuclear staining by performing the experiments on two different occasions resulting in four different readings to evaluate the extent of intensity for PKM2 immunostaining by viewing the specimens at 200 x magnification to score percent positive fields in a blinded fashion for statistical significance. PKM2 intensity scores of ≥ 2+ were considered positive, while ≤ 1+ were considered negative. We observed a very high concordance (exact 95% CI = 72.8–91.3) among the four sets of readings in both data sets (83.3%) obtained from each independent experiment, where scores 3+ and 4+ were combined as 3+, while scores 1+ and no score (value ±) were considered as 1+. These results demonstrate high precision in the IHC measurement for cytoplasmic and nuclear staining. For the remainder of the analysis, discordant scores were resolved by assigning the majority score and when there was an equal number of discordant scores, the lower score was used. For the rest of the analysis, all discordant cases in PKM2 were coded as negative.

**Fig 7:**
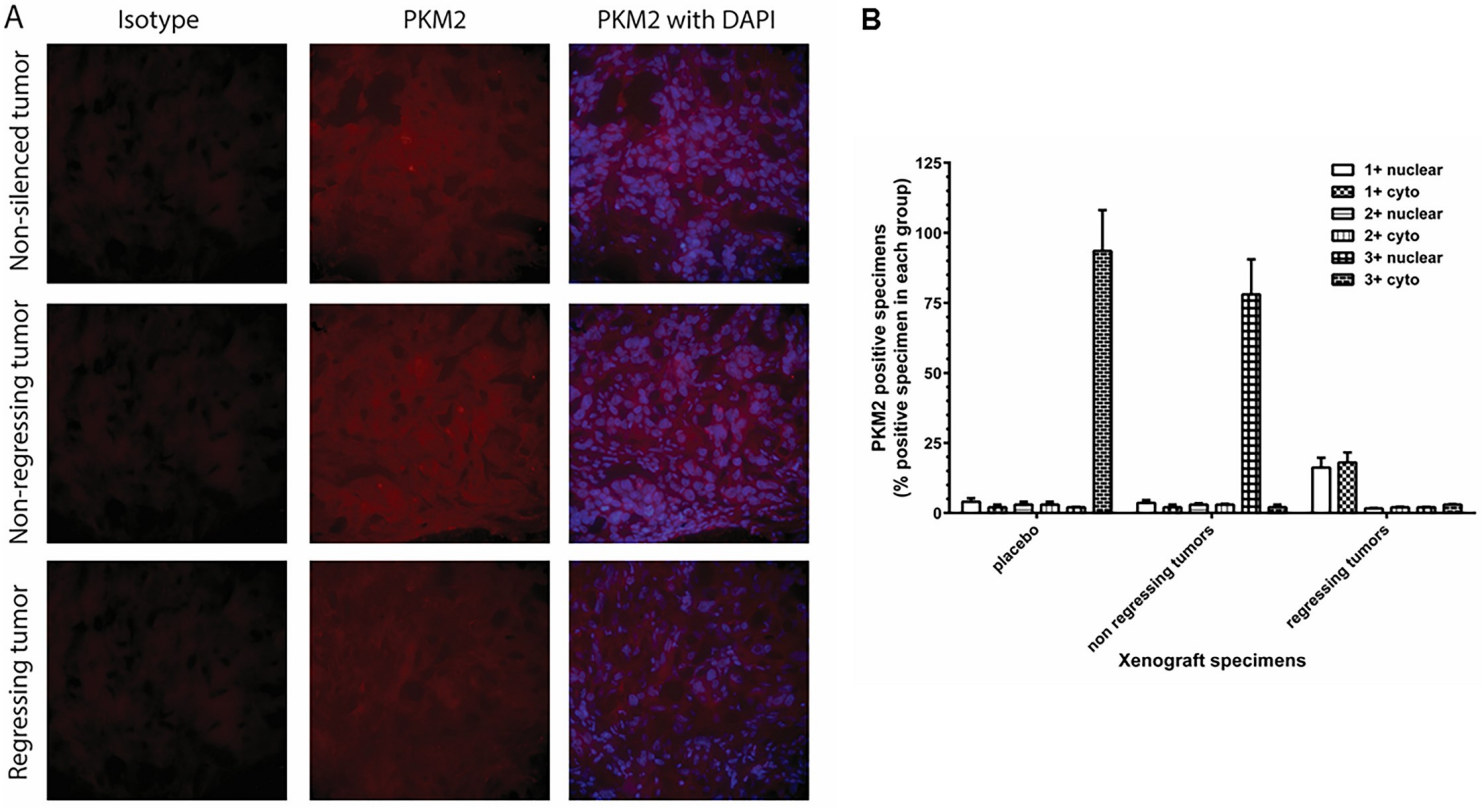
PKM2 expression in NSCLC tumor specimens. (A) H358 cells (non-silenced, mock-transfected and *shRNA-PKM2*) were implanted subcutaneously in nude mice and the tumor growth of each mouse was monitored. The mice were euthanized on day 80 and the s.c. tumors were surgically excised. The tumor specimens were fixed with 4% paraformaldehyde, embedded in OCT, cryo-sectioned, and immunostained with anti-PKM2 antibody. PKM2 was stained with Alexa 594 (red) and DAPI was used for nuclear staining (blue). The sections were viewed in Nikon fluorescence microscope at 200 x magnification. (B) Extent of PKM2 immunostaining in cytoplasm and nucleus was evaluated as ≤1+ negative; 2+ positive; 3+ strongly positive. % positive of total number of tumor cells was shown as mean ± SD.

Nuclear PKM2 was identified as a merged immunostaining of PKM2 (red) with DAPI (blue) staining as described in materials and methods. As shown in Fig 7A, tumors responding to *shRNA-PKM2* therapy demonstrated a significant decrease in cytosolic PKM2 in *shRNA-PKM2* treated mice xenografts (regressing) compared to parental or non-responding tumor xenografts (non-regressing) (P≤0.01). Immunostaining for nuclear PKM2 levels was ≤1+ with ~10% of the positive fields in most of the regressing tumors (Fig 7B). In contrast, the extent of IHC immunostaining in non-regressing xenografts showed a significant increase (3+) in nuclear PKM2 and reduced levels of cytosolic PKM2 compared to regressing or corresponding control (non-silenced) xenografts (P≤0.01). Interestingly, cytoplasmic PKM2 immunostaining intensity in non-regressing xenografts was lower (≤ 1+) in ~5% of the specimens and higher in nucleus (3+) in ≥ 75% than those seen in regressing tumor xenografts (P≤0.05; Fig 7B). But, >90% specimens from non-silenced control group showed 3+ cytosolic PKM2 expression. In the mice with a therapeutic response to SMI or *shRNA-PKM2* therapy, a sum of the positive values of PKM2 (2+ and 3+ combined) showed a statistically significant trend (non-silenced = 7% nuclear with 1+ and 88% cytoplasmic with 3+, non-regressing tumors = 75% nuclear with 3+ and 9% cytoplasmic with 1+, regressing tumors = 17.7% nuclear staining with 1+ and 12.56% cytoplasmic with 3+ PKM2 staining; exact P ≤ 0.00012) compared to negative values of PKM2 (≤ 1+) (Fig 7B).

## Discussion

We have demonstrated overexpression of PKM2 in patient-derived and established NSCLC cell lines. These cell lines not only overexpressed PKM2 protein but also high PKM2 enzyme activity in >55% of the cells with > 2+ immunostaining intensity. The overexpressed PKM2 in NSCLC is inhibitable in a concentration-dependent manner with either a drug or gene silencing by shRNA *in vitro*. These treatments affected both PKM2 enzyme activity and mRNA transcription, which subsequently led to reduced levels of PKM2 protein, as demonstrated by Western blot analysis. In contrast, three normal lung cell lines, which showed lower enzyme activity and mRNA, did not show such a dramatic response to both types of anti-PKM2 strategies. Further, we observed that PKM2 targeting with SMI or *shRNA-PKM2* significantly affected cell viability in all NSCLC cell lines and impaired the ability to form colonies *in vitro*, suggesting that PKM2 enzyme activity is important for tumor growth in NSCLC cells. The normal lung fibroblast cell lines failed to show such changes after targeting PKM2. Previously published results have shown that PKM2 can interact with mutant EGFR and HSP90 and prolonged the protein half-life of EGFR in lung cancer cells [14]. It is also reported that PKM2 supports tumor growth through regulating the expression of gene that involved in cell proliferation, migration and apoptosis [26]. Our present data have demonstrated a unique role of PKM2 as its enzyme inhibition by either SMI or gene silencing can influence the tumor growth *in vitro* and *in vivo*.

Elevated PKM2 is also associated with disease aggressiveness and poor overall survival of patients with signet ring cell gastric cancer [34], gall bladder cancer [35, 36], and pancreatic cancer [37]. Several published reports have shown that the deregulation of glucose metabolism and overexpression of PKM2 are key regulators of aerobic glycolysis, also known as the Warburg effect *in situ* in the tumor microenvironment [6, 38, 39]. This may be a consequence of the primary function of PKM2, which is to catalyze the phosphorylation from phosphoenol pyruvate to pyruvate at the last step of glycolysis, leading to generation of ATP needed for tumor growth [40, 41]. Cortes-Cros et al., demonstrated that knocking down of *PKM2* in colon carcinoma cell lines led to a decrease in PKM2 activity and an increase in the pyruvate kinase substrate phosphoenol pyruvate, leading to an increased macromolecular biosynthesis required for rapid proliferation; suggesting that PKM2 influences the metabolic state of the cells and has a role in tumor maintenance and growth *in vivo* [42, 43]. In a recent study of nodular hypoplasia formation in Upk2-HRAS transgenic mice, overexpression of PKM2 was identified as the principal tyrosine-phosphorylated protein in the genesis of urothelial carcinomas [44]. PKM2 is also involved in cancer initiation and progression [19, 29].

Our data demonstrated that targeting PKM2 with SMI and *shRNA-PKM2* affected cell viability and clonogenicity of NSCLC cells *in vitro*, which confirmed our results that PKM2 is a growth supportive factor We further investigated the mechanism for decreased cell viability and clonogenicity in PKM2 targeted NSCLC cells. We observed that SMI and *shRNA-PKM2* both downregulated PKM2 mRNA and decreased PKM2 activities, which correlated inversely with the number of apoptotic nuclei in targeted tumor cell populations. These changes are also associated with increased Caspase 3/7 activities in NSCLC cells upon treating them with SMI or *shRNA-PKM2*, which is a hallmark of apoptosis suggesting that SMI or *shRNA-PKM2* affected the cell viability by inducing Caspase 3/7 mediated apoptosis. Similar observations have been made by Goldberg and Sharp, who showed that targeting PKM2 with encapsulated siRNAs resulted in decreased cell viability and increased apoptosis in 10 members of the NCI-60 panel representing multiple types of cancer cell lines while having little effect in normal fibroblasts or endothelial cells and tumor regression in vivo mouse model [20]. It was also shown that *shRNA-PKM2* induced apoptosis *in vitro* and *in vivo* human lung cancer xenografts treated with docetaxel, which suggested that PKM2 could, to some extent, reverse chemotherapy resistance to cancer therapy [45, 46].

Interestingly, we identified a distinct subcellular compartmentalization of PKM2 enzyme activity and protein expression in cytoplasm and nucleus after targeting PKM2 by SMI or *shRNA-PKM2 in vitro*. We observed that PKM2 targeting by both strategies had decreased cytoplasmic and increased nuclear PKM2 enzyme activity in patient-derived established NSCLC cell lines, which also corroborated with PKM2 expression examined by ICC assay *in vitro*. Importantly, our *in vivo* mouse results also demonstrated such compartmentalization, which could differentiate animals responding to the PKM2 targeted therapy. Interestingly, the regressing xenografts had lower PKM2 in cytoplasm and nucleus while non-regressing tumor specimens showed a significant and dramatic increase in nuclear PKM2.

Despite its anti-proliferative activities *in vitro*, PKM2 inhibitor did not mediate significant effects on *in vivo* tumor growth in two NSCLC tumor xenografts and did not show statistically significant improvement of survival compared to control mice. The reason for lack of SMI activity *in vivo* is not clear. It is possible that PMK2 inhibitor levels, being a small molecular weight compound of 231.25 Da, were not retained in the tumor bed at a high enough level to exert a measurable anti-tumor effect. In contrast, PKM2 was silenced robustly by *shRNA* in tumor cells before xenograft development, which showed significant anti-tumor effects compared to non-silenced and mock-transfected xenografts. Because gene silenced and mock-transfected xenograft bearing mice were sacrificed on day 120 per our Institutional Animal Care and Use Committee (ACUC) protocols, the survival curves were found non-significantly separated in mock-transfected and PKM2 silenced group mice were not significant. The percent survival in mock-transfected and non-silenced mice was also non-significant as non-silenced group mice received transfection reagent without any DNA as additional control compared to negative control DNA in mock-transfected group mice. Similar studies of silencing PKM2 in colorectal cancer cell lines modulated PKM2 to the nucleus in response to oxaliplatin treatment sensitivity and characterized chemoresistance [17]. It was also observed that intrinsic resistance to gefitinib, an EGFR-tyrosine kinase inhibitor, is positively correlated with nuclear PKM2 levels in colorectal cancer cells [18].

The significance of variable PKM2 subcellular distribution is not known. Our results show that when PKM2 is downregulated by SMI or gene silencing, subcellular compartmentalization of PKM2 occurs. It is also not known that other types of PKM2 inhibitors critically play a similar role in subcellular compartmentalization of PKM2 in NSCLC *in vitro* or *in vivo* models of different types of human cancers. It is likely that PKM2 can have limited role as a biomarker of therapeutic response in certain types of cancer with different pathological grade and clinical stage (TNM). Additional studies in larger sample size of cancer subjects with different pathological grade and clinical stage (TNM) can potentially establish its meaningful role as a biomarker of therapeutic response in clinical settings. Why PKM2 is compartmentalized is not clear. PKM2 has been reported to be localized in the cell nucleus as a dimer that functions as an active protein kinase; whereas, PKM2 in its tetrameric form is localized in the cytosol and is an active pyruvate kinase [15]. It is possible that PKM2 functions as a protein kinase when is downmodulated by SMI. Additional studies are needed to unravel the significance of this compartmentalization. However, subcellular compartmentalization of PKM2 may have a role as a biomarker of response in distinguishing groups with therapeutic response versus no response when they were administered with anti-PKM2 therapy.

## Supporting information

S1 Fig Extent of immunostaining intensity for PKM2 expression in NSCLC cell lines. Immunocytochemical assay was performed to evaluate the intensity of PKM2 immunostaining in NSCLC cells using rabbit monoclonal anti-PKM2 antibody and alexa 595 conjugated goat anti rabbit IgG antibody. The immunostained cells were viewed at 200X magnification.
(TIF)

S1 Table Immunocytochemical (ICC) analysis for PKM2 in NSCLC cell lines. Extent of ICC was evaluated as ≤ 1+, weakly positive; 2+, strongly positive; 3+ stronger;4+, strongest. Percent positive field was counted after viewing PKM2positive cells at 200X magnification and shown in the parenthesis.
(DOC)

S2 Table ICC analysis of PKM2 in PKM2 targeted NSCLC cell lines. Extent of ICC was evaluated as ≤ 1+, weakly positive; 2+, strongly positive; 3+ stronger;4+, strongest. Percent positive field was counted after viewing PKM2positive cells at 200X magnification and shown in the parenthesis.
(DOCX)

S3 Table Analysis of subcellular compartmentalization of PKM2 in PKM2 targeted NSCLC cell lines. Extent of ICC was evaluated as ≤ 1+, weakly positive; 2+, strongly positive; 3+ stronger;4+, strongest. Percent positive field was counted after viewing PKM2positive cells at 200X magnification and shown in the parenthesis.
(DOC)

## Abbreviations

FBP: fructose-1,6-bisphosphate
NSCLC: non-small-cell lung cancer
PEP: phosphoenol-pyruvate
PKM2: pyruvate kinase M2
