# Supplementary figures and images for: Subcellular compartmentalization of PKM2 identifies anti-PKM2 therapy response in vitro and in vivo mouse model of human non-small-cell lung cancer
Source: PLoS One. 2019 May 23;14(5):e0217131. doi: 10.1371/journal.pone.0217131 (PMC6532891; doi:10.1371/journal.pone.0217131)

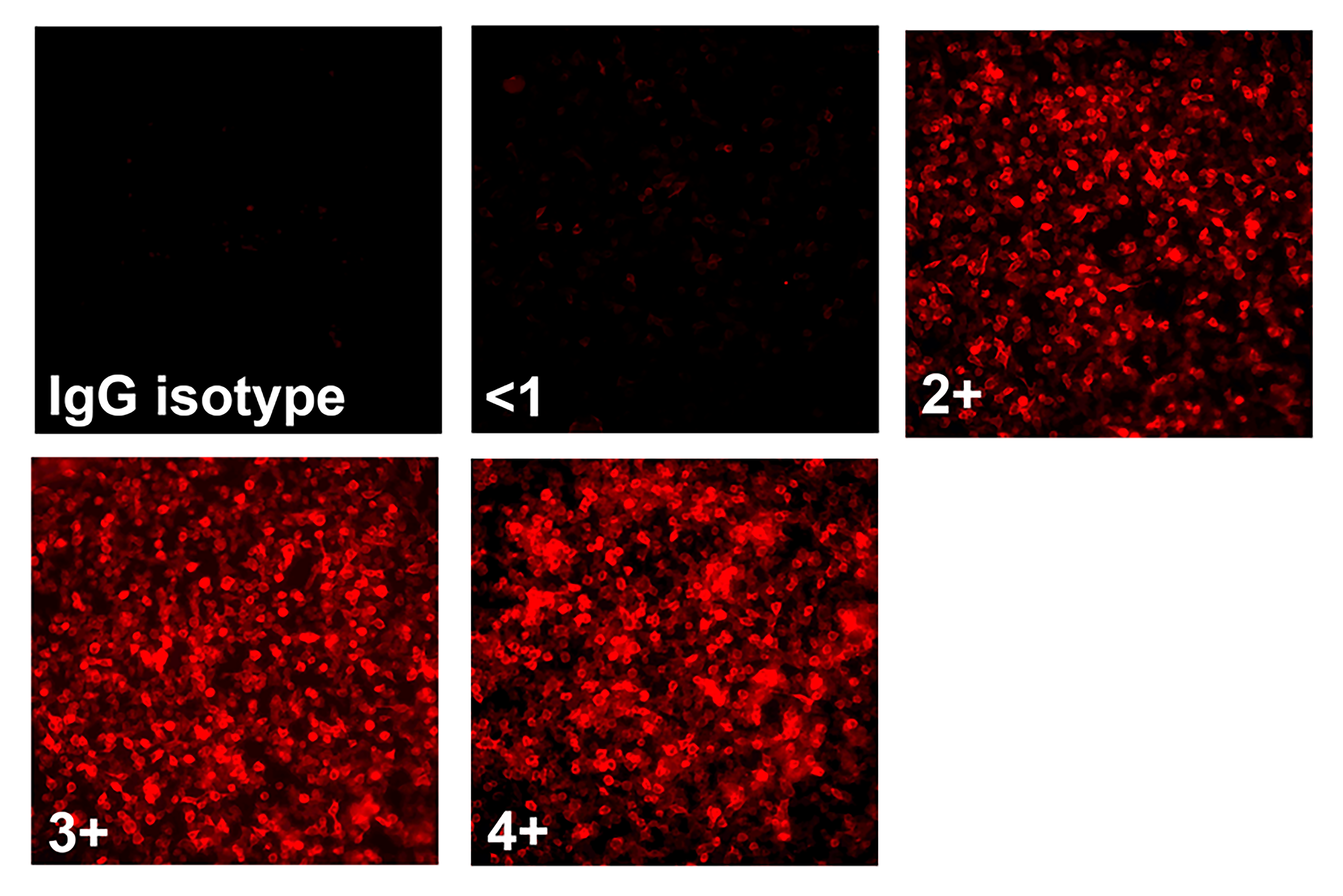

Supplement: S1 Fig — Immunocytochemical assay was performed to evaluate the intensity of PKM2 immunostaining in NSCLC cells using rabbit monoclonal anti-PKM2 antibody and alexa 595 conjugated goat anti rabbit IgG antibody. The immunostained cells were viewed at 200X magnification. (TIF) [file pone.0217131.s001.tif]
