# Supplementary material for: Subcellular compartmentalization of PKM2 identifies anti-PKM2 therapy response in vitro and in vivo mouse model of human non-small-cell lung cancer
Source: PLoS One. 2019 May 23;14(5):e0217131. doi: 10.1371/journal.pone.0217131 (PMC6532891; doi:10.1371/journal.pone.0217131)
Supplement: S1 Table — Extent of ICC was evaluated as ≤ 1+, weakly positive; 2+, strongly positive; 3+ stronger;4+, strongest. Percent positive field was counted after viewing PKM2positive cells at 200X magnification and shown in the parenthesis. (DOC) [file pone.0217131.s002.doc]

**SI Table 1: Immunocytochemical (ICC) Analysis for PKM2 in NSCLC cell lines**

____________________________________________________________________________

PKM2 Expression

-------------------------------------------------------------------------------------------------

Extent of immunostaining % positive cells

______________________________________________________________________________

**Normal Lung cell lines**

WI 38 ≤ 1+ 12

HEL 299 ≤ 1+ 08

IMR 90 ≤ 1+ 10

**NSCLC Cell lines (primary)**

LT 23 3+ 68

LT 44 3+ 55

LT 46 2+ 60

LT 54 4+ 72

LT 24 3+ 74

LT 28 4+ 58

LT 39 2+ 78

LT 60 3+ 62

LT 30 4+ 78

**Immortalized NSCLC cell lines**

H 1299 4+ 82

H 358 4+ 86

Extent of IHC was evaluated as ≤ 1+, weakly positive; 2+, strongly positive; 3+ stronger;4+, strongest. Percent positive field was counted after viewing PKM2positive cells at 200X magnification and shown in the parenthesis
