# Supplementary material for: Subcellular compartmentalization of PKM2 identifies anti-PKM2 therapy response in vitro and in vivo mouse model of human non-small-cell lung cancer
Source: PLoS One. 2019 May 23;14(5):e0217131. doi: 10.1371/journal.pone.0217131 (PMC6532891; doi:10.1371/journal.pone.0217131)
Supplement: S2 Table — Extent of ICC was evaluated as ≤ 1+, weakly positive; 2+, strongly positive; 3+ stronger;4+, strongest. Percent positive field was counted after viewing PKM2positive cells at 200X magnification and shown in the parenthesis. (DOCX) [file pone.0217131.s003.docx]

**SI Table 2: ICC Analysis of PKM2 in PKM2 Targeted NSCLC cell lines**

**______________________________________________________________________________**

**Cell Line SMI *shRNA-PKM2* ------------------------------------------------------------------------------------------------------------**

**Pre Post Pre Post**

**Normal Lung cell lines**

WI 38 ≤ 1+, (12) ≤ 1+, (10) ≤ 1+ (09) ≤ 1+ (08)

HEL 299 ≤ 1+, (08) ≤ 1+, (07) ≤ 1+ (07) ≤ 1+ (06)

IMR 90 ≤ 1+, (10) ≤ 1,+ (08) ≤ 1+ (09) ≤ 1+ (09)

**NSCLC Cell lines (primary)**

LT 23 3+ (68) 1+ (18) 3+ (72) ≤ 1 (04)

LT 44 3+ (55) 1+ (06) 3+ (55) ≤ 1 (04)

LT 46 2+ (60) 1+ (12) 2+ (60) ≤ 1 (06)

LT 54 4+ (72) ≤ 1+ (15) 4+ (72) ≤ 1 (07)

LT 24 3+ (74) 1+ (22) 3+ (74) ≤ 1 (11)

LT 28 4+ (58) 1+ (20) 4+ (58) ≤ 1 (08)

LT 39 2+ (78) ≤ 1 (15) 3+ (78) ≤ 1 (06)

LT 60 3+ (62) 1+ (18) 3+ (66) ≤ 1 (05)

LT 30 4+ (78) ≤ 1 (06) 4+ (78) 1 (12)

**Immortalized NSCLC cell lines**

H 1299 4+ (82) 1+ (08) 4+ (82) ≤ 1 (11)

H 358 4+ (86) ≤ 1 (10) 3+ (86) ≤ 1 (07)

Extent of ICC was evaluated as ≤ 1+, weakly positive; 2+, strongly positive; 3+ stronger;4+, strongest. Percent positive field was counted after viewing PKM2positive cells at 200X magnification and shown in the parenthesis
