# Supplementary material for: Subcellular compartmentalization of PKM2 identifies anti-PKM2 therapy response in vitro and in vivo mouse model of human non-small-cell lung cancer
Source: PLoS One. 2019 May 23;14(5):e0217131. doi: 10.1371/journal.pone.0217131 (PMC6532891; doi:10.1371/journal.pone.0217131)
Supplement: S3 Table — Extent of ICC was evaluated as ≤ 1+, weakly positive; 2+, strongly positive; 3+ stronger;4+, strongest. Percent positive field was counted after viewing PKM2positive cells at 200X magnification and shown in the parenthesis. (DOC) [file pone.0217131.s004.doc]

**SI Table 3: Analysis of Subcellular Compartmentalization of PKM2 in PKM2 Targeted NSCLC cell lines**

**_____________________________________________________________________________**

**Cell Line SMI *shRNA-PKM2* ---------------------------------------------- --------------------------------------------------**

**Pre Post Pre Post**

**-------------------- ------------------ ----------------------- ----------------------------**

**cyto/nucl cyto/nucl cyto/nucl cyto/nucl**

**______________________________________________________________________________**

**Normal Lung cell lines**

**WI 38 ≤ 1+, (10)/ ≤ 1+, (10) ≤ 1+, (09)/ ≤ 1+, (08) ≤ 1+, (6)/ ≤ 1+, (4) ≤ 1+,(05)/ ≤ 1+,(04)**

**HEL 299 ≤ 1+, (06)/ ≤ 1+, (03) ≤ 1+, (03)/ ≤ 1+, (03) ≤ 1+, (03)/ ≤ 1+, (02) ≤ 1+, (04)/ ≤ 1+, (03)**

**IMR 90 ≤ 1+, (05)/ ≤ 1+, (04) ≤ 1+ (09)/ ≤ 1+, (09) ≤ 1+, (04)/ ≤ 1+, (04) ≤ 1+ (05)/ ≤ 1+, (04)**

**Primary NSCLC Cell lines**

**LT 23 3+, (68)/ ≤1+, (18) ≤1+, (22)/ 2+, (54) 3+, (76)/ ≤1+, (10) ≤1+, (12)/ 2+, (64)**

**LT 44 3+, (55)/ ≤1+, (06) ≤1+, (15)/ 3+, (64) 3+, (61)/ ≤1+, (10) ≤1+, (18)/ 3+, (74)**

**LT 46 2+, (60)/ ≤1+, (10) ≤1+, (20)/ 2+, (76) 2+, (60)/ ≤1+, (10) ≤1+, (10)/ 2+, (82)**

**LT 54 4+, (72)/ ≤ 1+, (10) 1+, (22)/ 3+, (67) 4+, (72)/ ≤ 1+, (10) 1+, (17)/ 4+, (60)**

**LT 24 3+, (74)/ ≤1+, (08) ≤1+, (14)/ 3+, (61) 3+, (64)/ ≤1+, (12) ≤1+, (10)/ 3+, (86)**

**LT 28 4+, (58)/ ≤1+, (08) 1+, (28)/ 3+, (78) 4+, (64)/ ≤1+, (06) 1+, (20)/ 4+, (70)**

**LT 39 2+, (78)/ ≤1+, (05) ≤1+ (18) / 2+, (76) 2+, (56)/ ≤1+, (16) ≤1+ (28) / 2+, (80)**

**LT 60 3+, (62)/ ≤1+, (08) 1+, (18)/ 3+, (66) 3+, (69)/ ≤1+, (10) 1+, (10)/ 3+, (62)**

**LT 30 4+, (78)/ ≤ 1 (06) ≤1+ (28)/ 3+, (52) 4+, (70)/ ≤ 1 (08) ≤1+ (18)/ 4+, (68)**

**Immortalized NSCLCcell lines**

**H 1299 4+, (82)/ ≤1+, (08) ≤1+, (12)/ 3+, (71) 4+, (72)/ ≤1+, (06) ≤1+, (16)/ 3+, (81)**

**H358 4+, (86)/ ≤1+, (05) ≤1+, (15)/ 3+, (80) 4+, (80)/ ≤1+, (11) ≤1+ , (11)/ 4+, (88)**

**_____________________________________________________________________________________________**Extent of ICC was evaluated as ≤ 1+, weakly positive; 2+, strongly positive; 3+ stronger;4+, strongest. Percent positive field was counted after viewing PKM2positive cells at 200X magnification and shown in the parenthesis
